# Fellows as teachers: a teaching curriculum at a Veterans Affairs Medical Center

**DOI:** 10.5116/ijme.5edd.f8ab

**Published:** 2020-07-11

**Authors:** Tirsa M. Ferrer Marrero, Yan Zhou, Toni-Denise Espina, Rasika Chepuri, Amy Pluskota, Vijaya Ramalingam

**Affiliations:** 1 Department of Medicine, Division of Pulmonary and Critical Care Medicine, Medical College of Wisconsin, Milwaukee, WI, USA; 2 Clement J. Zablocki VA Medical Center, Milwaukee, WI, USA

**Keywords:** Fellows, teachers, teaching curriculum, Veterans Affairs Medical Center

## To the Editor

The medical literature supports the premise that the development of teaching skills should start as early as possible to enhance the vital role of physicians as teachers.^1^ Curriculums created to strengthen fellows' teaching skills could positively influence their career pathway.^2^^,^^3^ Furthermore, the exposure to clinical education may help the fellows in improving their communication abilities, their leadership, and their medical knowledge.^2^ Fellows are in a unique position to be effective educators because they may have more insight into trainees' learning needs.^2^ Woodfield and colleagues^4^ found that 100% medical students believed fellows' teaching was extremely helpful, 89% preferred fellows' teaching over consultants' and felt fellows' teaching was better suited for their level of training. Despite growing evidence supporting "Fellows as Teachers," programs aimed at strengthening fellows' teaching skills are available in only a minority of fellowship programs.^2^ Also, the majority of fellows' teaching opportunities occur in the inpatient consultation setting, which differs qualitatively from other scenarios, like the intensive care unit, outpatient clinic and simulation centers.^5^^,^^6^

Our academic institution has a partnership with the Clement J. Zablocki Veterans Affairs Medical Center. The medical center contains a fully-equipped state-of-the-art simulation center with simulation trainer models and ultrasound machines. This allows us to provide teaching sessions on ultrasound-guided vein cannulation to trainees and advanced practice providers.

After months of attending-only taught sessions on ultrasound-guided vein cannulation at the simulation center, a curriculum was created for the Pulmonary and Critical Care Medicine fellows to become the teachers of the sessions. The curriculum included a formal in-service program for the fellows to learn their roles and responsibilities as educators. During the in-service program, the fellows learned how to assess the trainees' needs and how to adapt their coaching to help to close any knowledge gaps. After the in-service was given, we established the fellow-taught sessions, and they were integrated into the monthly academic schedule. The fellow-taught sessions eventually substituted the attending-taught sessions. A faculty member observed the fellow-taught sessions and provided feedback to improve the fellows' teaching competencies. The Veterans Affairs Medical Center Senior IRB Analyst approved this project. In this letter, we report our experience with implementing the curriculum of fellows as teachers of ultrasound-guided vein cannulation. We also provide details about the trainees' overall experience during the teaching sessions.

We conducted the ultrasound-guided vein cannulation teaching sessions 1-2 times a month, at the Clement J. Zablocki Veterans Affairs Medical Center simulation center. During the sessions, the focus was on teaching ultrasound basics, image acquisition, identification of major veins, review of key anatomical structures, and advancing the needle into the center of the vein. The sessions’ participants were internal medicine residents and interns, medical students, and advanced practice providers who filled out an eight-point total pretest before the session, and an eight-point total posttest afterwards. The participating trainees also filled out an anonymous and voluntary evaluation. The pretest and posttest were similar and were created to explore three specific domains. The domains were: (i) recognition of the vessels and surrounding structures under ultrasound; (ii) identification of the ultrasound views that will assure a safe and successful central vein cannulation; (iii) the proper use of knobology to assure successful peripheral vein cannulation. The test contained two multiple-choice questions, one true or false, and one question asking for the identification of the structures in an ultrasound image.

Seventy-four trainees in total participated in the sessions. Fifty-four (73%) were residents or interns, 17(23%) Medical Students, 2(3%) Advanced Practice Providers, and 1(1%) did not answer. Twenty-four (32%) trainees participated in the fellow-taught sessions and fifty (68%) in the attending-taught sessions. There was a higher proportion of medical students in the fellow-taught sessions (38%) compared to the attending-taught ones (16%). There was a trend to higher trainees' posttest scoring in both groups. However, the improvement in the posttest scoring was more pronounced and significant in the fellow-taught group. In the fellow-taught group, nineteen out of twenty-four participant trainees (79%) completed the session's evaluation.  All the trainees subjectively felt that fellows' teaching was effective, and 89% of them thought the fellows' teaching positively influenced their education.

Although there was a higher proportion of medical students in the fellow-taught group, the group had a more significant improvement in the posttest scores. This finding attests to fellows' ability to deliver high-quality education to learners at different levels of training. As Woodfield and colleagues^4^ showed, medical students often believe that fellows' communication skills are more useful in education compared to the consultants. Furthermore, the fellows are more likely to be in touch with the students' educational needs providing a more peer-like assisted learning in a less threatening and more sympathetic environment.^4^

Thus far, our experience with the implementation of this curriculum has been very positive. The curriculum is giving the fellows additional exposure to clinical education in an environment other than inpatient consults. Most of the fellows' teaching opportunities that have been published in medical literature occur in the inpatient consultation setting.^5^^,^^6^ On the other hand, our project involves a fellows' teaching opportunity in the setting of simulation-based education; this contributes to the uniqueness of our report. The main strength of this curriculum is the design, which could be easy to implement at any teaching hospital with a simulation center.  Other contributing factors to our positive results include but are not limited to the dedicated curriculum created by our division, the availability of resources at the Veterans Affairs Medical Center simulation center, and the institution of a formal in-service program for the fellows.

The lack of opportunities for fellows to develop as teachers is at odds with the multitude of residents-as-teachers' programs available, and their role as teachers may be underrecognized.^2^ As shown in this report, fellows teaching has a positive influence on trainees', highlighting the vital role that fellows have as teachers.^3^^,^^5^ In this letter we show that fellows' teaching in a simulation center and using simulation-based education can be beneficial.^7^ Although our data is limited to the trainees' test results and evaluations, the information that we have gathered reflects that the fellows' teaching is not only effective but is also highly appreciated by the trainees.

## References

[1] Chen HC, Wamsley MA, Azzam A, Julian K, Irby DM, O'Sullivan PS (2017). The health professions education pathway: preparing students, residents, and fellows to become future educators.. Teach Learn Med.

[2] Miloslavsky EM, Boyer D, Winn AS, Stafford DE, McSparron JI (2016). Fellows as teachers: raising the educational bar.. Ann Am Thorac Soc.

[3] Horn L, Tzanetos K, Thorpe K, Straus SE (2008). Factors associated with the subspecialty choices of internal medicine residents in Canada.. BMC Med Educ.

[4] Woodfield G, O'Sullivan M (2014). Clinical teaching fellows: everyone's a winner.. Clin Teach.

[5] Miloslavsky EM, McSparron JI, Richards JB, Puig A, Sullivan AM (2015). Teaching during consultation: factors affecting the resident-fellow teaching interaction.. Med Educ.

[6] Chen DC, Miloslavsky EM, Winn AS, McSparron JI (2018). Fellow as clinical teacher (FACT) curriculum: improving fellows' teaching skills during inpatient consultation.. MedEdPORTAL.

[7] Bearman M, Greenhill J, Nestel D (2019). The power of simulation: a large-scale narrative analysis of learners' experiences.. Med Educ.

